# Design of Self-Healing EPDM/Ionomer Thermoplastic Vulcanizates by Ionic Cross-Links for Automotive Application

**DOI:** 10.3390/polym14061156

**Published:** 2022-03-14

**Authors:** Woo Seok Jin, Pranabesh Sahu, Sung Min Park, Jun Ha Jeon, Nam Il Kim, Jae Hyeon Lee, Jeong Seok Oh

**Affiliations:** 1 Department of Materials Engineering and Convergence Technology, ReCAPT, Gyeongsang National University, 501, Jinju-daero, Jinju 52828, Korea; qkswldmlgla@gnu.ac.kr (W.S.J.); pranab91@gnu.ac.kr (P.S.); 2 With Advanced Passion & System, WAPS Co., Ltd., 8F, 45, Centum dong-ro, Haeundae-gu, Busan 48059, Korea; smpark@waps.com; 3 Industrial Materials Research Center, Korea Institute of Footwear & Leather Technology, 152, Danggamseo-ro, Busanjin-gu, Busan 47154, Korea; juna@kiflt.re.kr; 4 Energy Materials R&D Center, Korea Automotive Technology Institute, Cheonan 31214, Korea; nikim@katech.re.kr (N.I.K.); leejh@katech.re.kr (J.H.L.)

**Keywords:** thermoplastic vulcanizates, ionomer, ionic cross-linking, mechanical properties, self-healing

## Abstract

The development of smart elastomeric materials with inherent self-repairing abilities after mechanical damage has important technological and scientific implications, particularly in regard to the durability and life cycle of rubber products. The interest in self-healing materials for automotive applications is rapidly growing along with the increasing importance of vehicle scratch quality and quantity. The creation of a reversible network by noncovalent ionic cross-linking in elastomer/rubber blends is an effective approach to generate the self-healing phenomenon, with reprocessing and recycling properties. In this work, thermoplastic vulcanizates (TPVs) were prepared using ethylene–propylene–diene (EPDM) polymers and high-acid-containing thermoplastic ionomers. Along with the general EPDM, maleic anhydride grafted EPDM (EPDM-*g*-MAH) was also used for the preparation of the TPVs. The strategy was based on a simple ionic crosslinking reaction between the carboxyl groups present in the ionomer and zinc oxide (ZnO), where the formation of reversible Zn^2+^ salt bondings exhibits the self-healing behavior. The heterogeneous blending of EPDM and ionomers was also used to investigate the thermal and mechanical properties of the TPVs. The experimental findings were further supported by the surface morphology of the fracture surfaces viewed using microscopy. The self-healing behavior of the TPVs has been identified by scratch resistance testing, where the EPDM-*g*-MAH TPVs showed excellent healing efficiency of the scratch surface. Therefore, this work provides an efficient approach to fabricate new ionically cross-linked thermoplastic vulcanizates with excellent mechanical and self-repairing properties for the skins of automotive interior door trims and instrument panel applications.

## 1. Introduction

Rubbers are extensively used in various fields, especially in vehicle tires and different automotive parts. Due to increased road accidents, scratches on automotive parts occur quite often, decreasing the durability and life cycle of the products. Self-healable rubber is expected to contribute to the advanced manufacturing of different automobile parts [1,2]. The concept of developing self-healable materials is highly motivating in rubber technology and engineering [3,4]. In recent years, self-healing elastomers for automotive application have attracted tremendous attention for improving the structural reliability and lifetime of the material with respect to damage tolerance [5,6,7,8]. Self-healing is defined as the ability of a material to recover or repair from the suffered physical or mechanical damage automatically, or with the aid of an external stimulus (temperature, pressure, and chemicals). Different approaches have been formulated to engineer a polymeric material or composites with the capability to self-heal [9,10,11]. Conventional vulcanized rubbers do not show self-healing behavior, due to their irreversible crosslinked network structure. In order to develop self-healing properties, dynamic crosslinks, or physical associations, are used to construct a reversible network in rubbers [11,12,13,14]. Previously, a wide range of self-healing elastomers were developed with conventional rubbers and thermoplastic elastomers (TPEs) using noncovalent interactions, such as hydrogen bonding, ionic bonding, and reversible covalent bonding, such as the Diels–Alder reaction [15,16,17,18]. However, there are no such significant studies found in the field of self-healing thermoplastic vulcanizates (TPVs). TPVs are widely used in the rubber industry due to their combination of high performance and recyclability of thermoplastics. From a sustainability point of view, there is also an urgent need to develop self-healable and recyclable TPVs.

Until now, self-healing research has been predominantly focused on polymeric systems that heal through chemical means. The presence of ionic associations or crosslinking in rubbers and ionomers has also been noted as a powerful approach for designing self-healing rubbers [19,20,21]. Xu et al. [22] developed an ionic cross-linked supramolecular hybrid network with natural rubber using zinc dimethacrylate, where the reversible nature of ionic associations exhibited self-healing qualities. The ionomer properties vary depending on the polymer backbone, ion content, and degree of neutralization. The ionomer behaves as an elastomer at room temperature; whereas, it can be processed as thermoplastic at a higher temperature. The reversibility of ionic associations facilitates the healing phenomenon to allow a cut sample to regain its original properties [23,24]. These ionic polymers or ionomers can be controllably vulcanized with commercial rubbers to form ionic networks for developing novel materials with self-healing behaviors [4]. Xiang et al. [11] fabricated a self-healable vulcanized chloroprene rubber using an organic complex copper (II) methacrylate (MA-Cu) catalyst at elevated temperature. The inherent sulfur cross-links was reversibly exchanged at 120 °C, reflecting excellent reshaping, self-healing, and recycling properties in the vulcanizates. In this context, the fabrication of TPVs with self-healing properties for smart application is highly desired. 

In the present work, ionomer/EPDM composites were prepared through the dynamic vulcanization process. Along with the general EPDM rubber, maleic anhydride-grafted-EPDM rubber was also used. The thermal and mechanical properties, along with the compression set of the composites, were investigated. The surface morphology of the vulcanizates was also explored. The self-healing behavior of thermoplastic vulcanizates has also been identified by a scratch-resistance test. The developed material is expected to be applied to the skins of automotive door trims and for instrument panel applications. From the viewpoint of sustainable development, this efficient strategy of constructing reversible dynamic crosslinks in a solid state shows a good prospect for the rational design of EPDM rubbers for perceived applications in the automobile industry. 

## 2. Material and Methods

### 2.1. Materials 

In this research, ethylene–propylene–diene rubber (EPDM) grade KEP570P with an ethylene content of 70 wt%, Mooney Viscosity ML (1 + 4) at 125 °C of 53, and ethylene–propylene–diene monomer-grafted maleic anhydride (EPDM-*g*-MAH), grade KEPA1150, in pellet form, were purchased from Kumho Petrochemical Ltd., Daejeon, Korea. Ionomer (HA60D) grade High acid 60D, partially neutralized with zinc, was supplied by WAPS Co., Ltd., Busan, Korea. The carboxylic acid (acrylic acid) content of the ionomer is 20% and the degree of neutralization is 50%. Reagent grade zinc oxide and stearic acid were obtained from Daejung Chemicals & Metals Co., Ltd., Seoul, Korea. The 2-2′-Dithiobis (benzothiazole) (MBTS), tetramethylthiuram disulfide (TMTD), and sulfur were procured from Sigma-Aldrich, Seoul, Korea . All the compounding chemicals were used as received without further purification. 

### 2.2. Preparation of Thermoplastic Vulcanizates

TPVs of different compositions were prepared using the melt mixing technique in an internal mixer following the method in Table 1. The vulcanization and formulation conditions were determined based on our recent published work on EPDM composites filled with carbon black [25]. The EPDM/HA60D and EPDM-*g*-MAH/HA60D relative concentrations (wt%) used for this study are 60/40 and 70/30. First, EPDM and Ionomer (HA60D) (60/40, wt%) were premixed at 130 °C and 60 rpm for 8 min. Then, the first set of compounding ingredients was added and mixed for another 2 min. Finally, sulfur was added to the mixture and further mixed for 8 min. EPDM-*g*-MAH/HA60D blends (60/40 and 70/30, wt%) were also prepared using the same mixing procedure. The compounds were kept at room temperature for 24 h before conducting the cure characteristics assessment. 

After the melt mixing, all the blends were compression molded and vulcanized at 170 °C for 10 min to obtain 2 ± 0.1 mm thick plain sheets.

### 2.3. Characterization Methods

Differential Scanning Calorimetry (DSC): Thermal analysis was carried out in a TA Instruments DSC Q20 (New Castle, DE, USA), under N_2_ atmosphere at 10 °C/min heating rate from −70 to 300 °C for all TPV blends and pure components. The glass transition temperature (*T_g_*) and melting temperature (*T_m_*) were taken as the inflection points from the heating curve to identify the heterogeneous phase structure of the TPVs compounds. 

Thermogravimetric (TGA) Analysis: The thermal degradation characteristics of pristine compounds and TPV blends were obtained using a TA Instruments DSC Q50 (New Castle, DE, USA) () under nitrogen atmosphere from room temperature to 600 °C at a heating rate of 10 °C/min.

Mechanical Properties: The tensile properties of the dumbbell-shaped specimens were measured in a Myungji Tech Tensometer 2000 (Yongsan-gu, Seoul, Korea) at a crosshead speed of 500 mm/min. The thickness of the specimens was kept as 2 ± 0.1 mm. The stress-strain curves of five samples were recorded and the average values are reported. 

Hardness and Compression Set: The hardness was evaluated using a Shore A durometer (Asker CL-150, Kobunshi Co., Kyoto, Japan) following ASTM D2240. The compression set (CS) % was measured with a Myungji Tech compression set (Yongsan-gu, Seoul, Korea) using cylindrical samples with a diameter of 29 ± 0.5 mm and a thickness of 12.5 ± 0.5 mm, according to ASTM D395. The samples were compressed using two parallel plates to a linear deformation of 25% at 70 °C for 22 h.

Microscopy Analysis: To elucidate the morphology of the TPVs, field-emission scanning electron microscopy (Seron Technology, AIS 2300C, Uiwang-si, Gyeonggi-do, Korea) was carried out at an accelerating voltage of 20 kV. A cryo-fractured sample from a tensile specimen was used for analysis. The samples were mounted on aluminum stubs with carbon tape and then sputtered with gold coating for observation.

Self-Healing Scratch Test: A self-healing test was performed using a scratch hardness tester (430P, Erichsen, Westlake, OH, USA). The scratches were created by applying a 0.50 mm diameter round ball-type tungsten carbide (WC) tip on the TPVs surface at a constant load (10 N) and speed (40 mm/s); then the sample was placed in a convection oven maintained at 89 °C for 24 h. The test method was followed according to BMW GS97034-9 for leather-type structured plastic materials for car interiors. The change of scratch width was monitored using the laser confocal microscope (OLS 5000, Olympus, Seocho-gu, Seoul, Korea) technique. The self-healing efficiency was calculated as follows:

(1) $$\;\;\;\;\;Healing\;Efficiency\;\left(\%\right)=\frac{\left(Initial\;depth-Final\;depth\right)}{\left(Initial\;depth\right)}\times 100\;\;\;\;\;$$

## 3. Results and Discussion

### 3.1. Fabrication of Thermoplastic Vulcanizates for Self-Healing Application

A number of blends, consisting of different contents of EPDM, EPDM-*g*-MAH, and HA60D, were dynamically vulcanized to fabricate self-healing TPVs. The EPDM-*g*-MAH polymer was used to investigate compatibility issues in the blends. It was observed that the EPDM-*g*-MAH showed better compatibility with the ionomer compared to the pristine EPDM. We also considered the effect of sulfur content on the mechanical properties of the EPDM-*g*-MAH/HA60D composites. 

**Figure 1:**
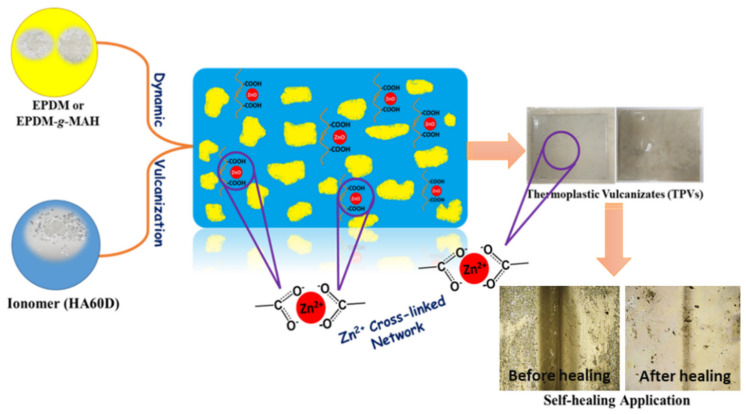
The schematic of the ionic cross-link formation in thermoplastic vulcanizates by Zn^2+^ salt-bondings with the carboxyl groups.

The strategy is based on the simple reaction between the acid groups of ionomer and zinc oxide in the TPVs, where the Zn^2+^ ions pairs self-aggregate to form an ionic cross-linked network. The schematic of the reaction and ionic cross-links formation in the thermoplastic vulcanizates is shown in Figure 1. These ionic cross-linking domains act as reinforcers to enhance the mechanical properties of the TPVs, and the reversible nature of the ionic network serves as an external stimuli to make TPVs recyclable and self-healable. 

### 3.2. Mechanical Properties

**Figure 2:**
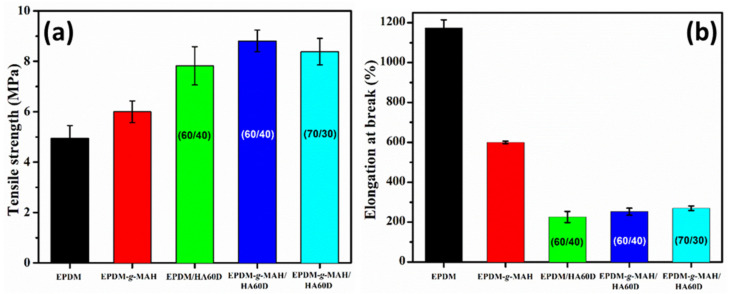
The mechanical properties of EPDM, EPDM-*g*-MAH, EPDM/HAD60, and EPDM-*g*-MAH/HAD60 TPVs: (**a**) The tensile strength plots, (**b**) The elongation at break plots.

The introduction of ionic crosslinking into a polymer blend is expected to improve the mechanical properties of the compounds. The interaction of the ionomer with ZnO in the composites was confirmed by the rise in mechanical strength of the TPVs. Figure 2a,b shows the tensile strength and elongation at break plot of the pristine material and the TPVs with various blend ratios. For comparison, the neat EPDM and EPDM-*g*-MAH mechanical properties were measured. By using the ionomer (HAD60) with EPDM in the TPVs, the tensile strength was increased from 4.9 MPa to 7.8 MPa. With EPDM-*g*-MAH/ HAD60 (60/40 wt%), the tensile strength increased from 6.0 MPa to 8.8 MPa. As expected, with the decrease in the ionomer content for the 70/30 wt% blend, there was a drop in tensile value. This significant improvement of tensile properties represents the formation of ionic cross-linking with the ionomer phase during the curing process. A higher TS value in the case of EPDM-*g*-MAH/ HAD60 TPVs might also indicate an improved interaction between the rubber phases and ionomer phase [26]. 

In addition, the pristine EPDM and EPDM-*g*-MAH exhibited a high elongation of 1170 and 600%, respectively. With the addition of the ionomer, the elongation at break decreases to 220% for EPDM/HAD60 (60/40) and to 250 and 270% for EPDM-*g*-MAH/HAD60 (60/40 and 70/30) blends.

**Figure 3:**
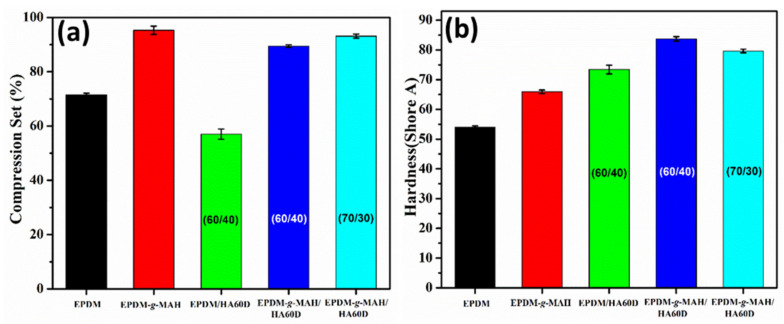
(**a**) The compression set properties and (**b**) the hardness plots of EPDM, EPDM-*g*-MAH, EPDM/HAD60, and EPDM-*g*-MAH/HAD60 TPVs.

To determine the effect of ionic crosslinks and ionomer loading in the TPVs, the compression set property was measured at 70 °C for 22 h. The compression set property provides an insight into the degree of ionic interaction with the acid group of the ionomer, as well as the ability of thermoplastic vulcanizates to retain their elastic properties after the prolonged action of compressive stresses. Figure 3a shows the compression set properties of the prepared TPVs. In the case of the neat EPDM and EPDM-*g*-MAH compounds, the compression set is higher, whereas with the addition of the ionomer in the corresponding vulcanizates, the compression set decreases. This could be due to the presence of higher crosslinks in the multilayered vulcanizates that resist deformation, thus improving the compression set property. 

Figure 3b shows the dependency of hardness on the ionomer content in the TPVs. The hardness of the neat EPDM and EPDM-*g*-MAH compounds was observed at about 54 and 66 Shore A. It could be clearly seen that the addition of HA60D to the compounds with same amount of filler loading yielded a significant effect on the hardness value. For the 60/40 EPDM and EPDM-*g*-MAH compounds, the hardness value increased to 73 and 84 Shore A, respectively. Higher crosslink density in the vulcanizates is responsible for higher hardness. Moreover, with the lowering of the EPDM-*g*-MAH/HAD60 (70/30) ionomer loading, the hardness value decreased.

**Figure 4:**
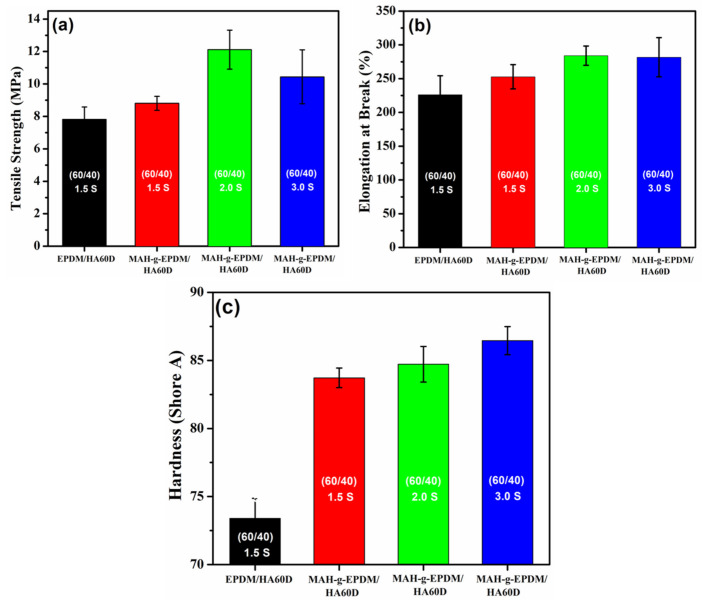
The mechanical properties of EPDM-*g*-MAH/HAD60 TPVs with different sulfur loadings: (**a**) Tensile strength, (**b**) Elongation at break and (**c**) Hardness plots.

Therefore, the presence of polar groups in EPDM-*g*-MAH provides the maximum effect of compatibilization with the ionomer, thus enhancing the mechanical properties of the vulcanizates. The mechanical strength results were used to represent the compatibility and the quality of the produced EPDM-*g*-MAH/HAD60 thermoplastic vulcanizates. In addition, the effect of the sulfur content (1.5 phr, 2.0 phr and 3.0 phr) on the mechanical properties of the TPVs was investigated. Figure 4a–c depicts the correlation between the TS, EAB, and hardness values and the amount of sulfur loading in the EPDM-*g*-MAH/HAD60 (60/40) TPVs. The TS increased to 12 MPa with 2.0 phr sulfur content and decreased to 10.4 MPa with the 3.0 phr sulfur compound. In addition, the vulcanizates showed increased hardness with increasing sulfur loading, as shown in Figure 4c. 

### 3.3. Thermal Analysis

**Figure 5:**
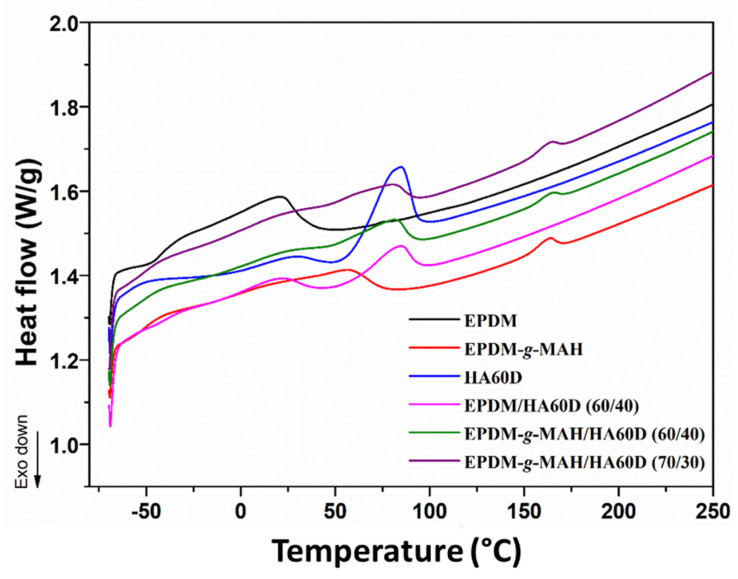
The DSC thermograms of the pristine compounds and the EPDM/HA60D, EPDM-*g*-MAH/HA60D vulcanizates.

DSC was used to investigate the thermal behavior of the self-healable thermoplastic vulcanizates. Figure 5 shows the DSC traces of the raw materials EPDM, EPDM-*g*-MAH, HAD60, and the vulcanizates. The glass transition temperature (*T_g_*) is an indirect representation of the heterogeneous nature of the polymeric vulcanizates. The results are summarized in Table 2. The *T_g_* of the neat EPDM and EPDM-*g*-MAH compounds were observed at −47 and −55 °C, respectively. In contrast, the melting point (*T_m_*) of the HA60D and EPDM-*g*-MAH compounds were found at +82 and 162 °C, approximately. The EPDM/HA60D (60/40) vulcanizate showed two distinct peaks (*T_g_* and *T_m_*) corresponding to the two constituents for the compositions of the blend. For the EPDM-*g*-MAH/HA60D vulcanizates, a single *T_g_* value and two different melting peaks were obtained: one well below room temperature, accordant with the rubber phase, and the other at about 80 °C, in line with the ionomeric phase, thus manifesting the heterogeneous phase structure of the composites. 

**Figure 6:**
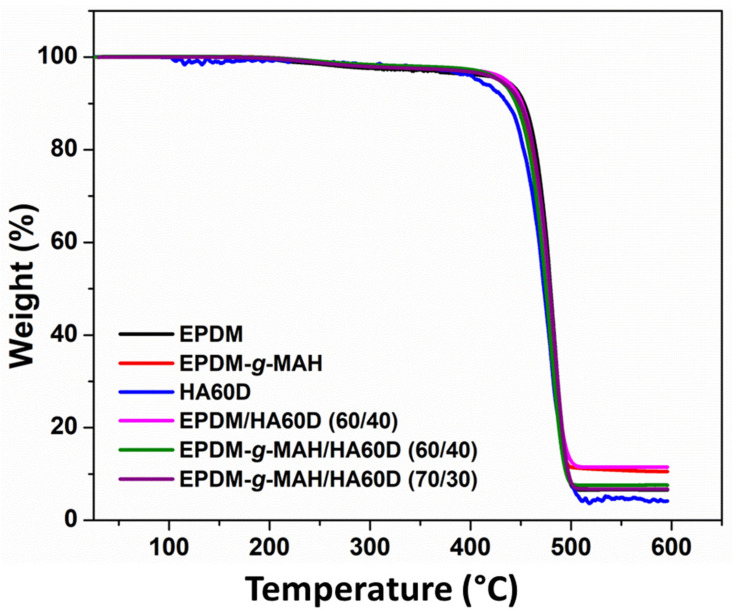
The thermogravimetric analysis of the pristine compounds and the EPDM/HA60D, EPDM-*g*-MAH/HA60D vulcanizates.

Figure 6 represents the thermogravimetric analysis of all the pristine and thermoplastic vulcanizates. The EPDM/HA60D and EPDM-*g*-MAH/HA60D vulcanizates showed higher thermal stability compared to the neat compounds. In addition, when comparing the effect of the EPDM and EPDM-*g*-MAH, it was observed that the maleic anhydride grafted EPDM provides a slightly better thermal stability to the vulcanizates.

### 3.4. SEM Analysis

**Figure 7:**
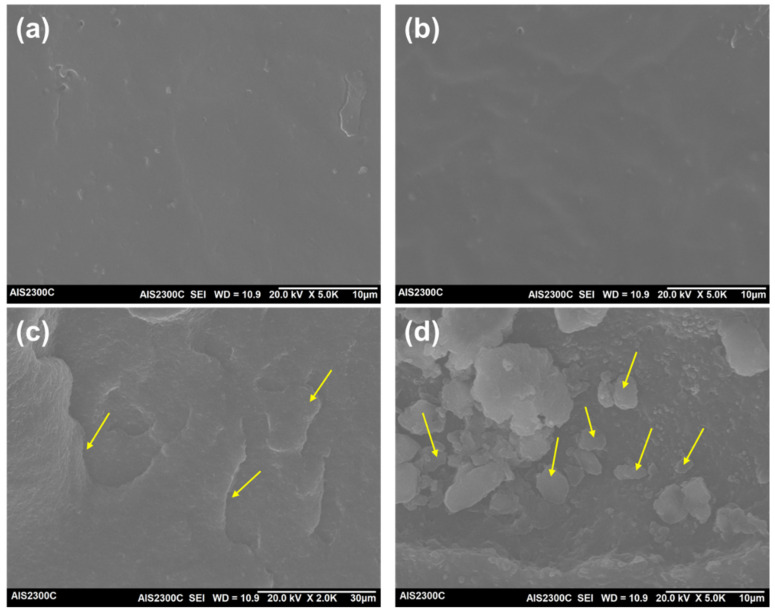
SEM images of the cryogenically fractured surface of the samples: (**a**) neat EPDM, (**b**) neat EPDM-*g*-MAH, (**c**) EPDM/HA60D TPVs, and (**d**) EPDM-*g*-MAH/HA60D TPVs.

To provide an intuitive observation of the inside structure of the thermoplastic vulcanizates, SEM imaging of the cryogenically fractured surface was performed. Figure 7 displays the SEM micrographs for the EPDM and EPDM-*g*-MAH compounds and the EPDM/HA60D and EPDM-*g*-MAH/HA60D vulcanizates. From the images (Figure 7a,b), we see only smooth surfaces, representing the EPDM phase. In the case of EPDM/HA60D TPVs (Figure 7c), we see co-continuous phase morphology, possibly due to the higher ethylene content of the EPDM grade, i.e., the semi-crystalline nature blended with the crystalline thermoplastic ionomer. However, for the dynamically cured EPDM-*g*-MAH/HA60D compound (Figure 7d), the morphology is the droplet/matrix type (indicated by arrows), with dispersed EPDM-*g*-MAH in the ionomer (HA60D) matrix. This is attributed to phase inversion during melt mixing, the common morphology observed in the dynamically cured blend of a thermoplastic and an elastomer.

### 3.5. Self-Healing Property

**Figure 8:**
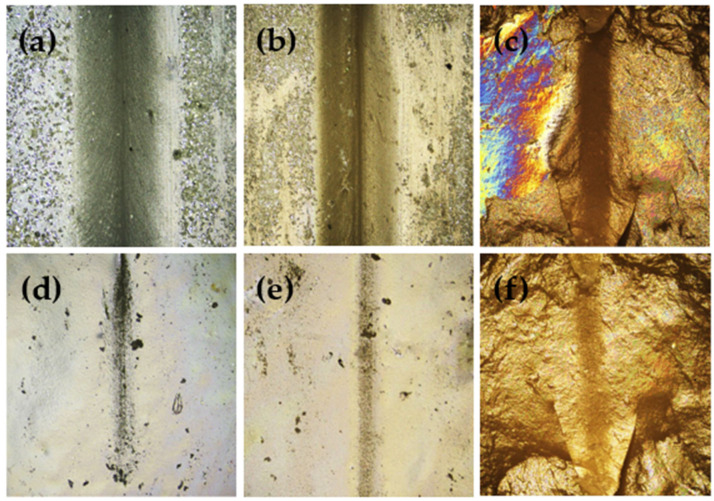
The optical micrographs of the EPDM-*g*-MAH/HA60D and EPDM /HA60D mixtures taken before (**a**–**c**) and after (**d**–**f**) thermal exposure for 24 h: (**a**) 60/40 EPDM-*g*-MAH/HA60D, (**b**) 70/30 EPDM-*g*-MAH/HA60D, (**c**) 60/40 EPDM/HA60D, (**d**) 60/40 EPDM-*g*-MAH/HA60D, (**e**) 70/30 EPDM-*g*-MAH/HA60D, (**f**) 60/40 EPDM/HA60D.

Figure 8a–e shows the optical micrographs of the EPDM-*g*-MAH/HA60D and EPDM/HA60D vulcanizates taken before and after thermal aging. An initial scratch with the width of around 300~500 μm gradually decreases to around 40–80 μm after thermal exposure at 89 °C and then becomes unclear after 24 h. The EPDM-*g*-MAH/HA60D mixtures exhibit a high scratch recovery rate above 87% within 24 h, regardless of the compositions (Figure 8d,e), while the scratch recovery rate of the 60/40 EPDM/HA60D mixture is difficult to determine (Figure 8f). It is noticed that the surface of the 60/40 EPDM/HA60D vulcanizate is uneven, with many ripple formations, and therefore, the width of the scratch is not uniform throughout the surface (Figure 8c,f). The self-healing recovery of the respective samples are tabulated in Table 3.

## 4. Conclusions

This works reports the preparation of self-healing thermoplastic vulcanizates based on a EPDM polymer and a thermoplastic ionomer (60/40 and 70/30 wt%) by melt-mixing. The study also found that the use of EPDM-*g*-MAH in the TPVs showed better compatibility with the ionomer, affecting the mechanical and thermal properties, as well as the self-repairing behavior, of the final material. The formation of ionic clusters with -COOH groups and ZnO in the blends provided improved mechanical properties and elasticity of the vulcanizates. Interestingly, the tensile strength and hardness values increased with the ionomer content in all the TPVs composition. In addition, the EPDM and EPDM-*g*-MAH/ionomer vulcanizates displayed an improved compression set property corresponding to their neat compounds due to the presence of higher crosslinks in the TPVs. The morphological investigation verified the dispersed rubber phase in the ionomer matrix for the EPDM-*g*-MAH/ionomer composites, whereas a co-continuous morphology was observed for the EPDM/ionomer vulcanizates. As a consequence of ionic-crosslinking and its reversible nature, both the EPDM-*g*-MAH/ionomer (60/40) and (70/30) thermoplastic vulcanizates showed good shape recovery of about 88% after surface-scratch healing. On the other hand, due to the uneven surface structure of the EPDM/ionomer vulcanizates, it was difficult to measure their scratch recovery performance. Finally, it can be concluded that the formation of ionic crosslinks in thermoplastic vulcanizates opens up new opportunities for the development of high performance and smart elastomeric materials. The present study hopes to open new avenues for the sustainable application of cross-linked rubber composites showing recyclable and healable properties, where the effects of the dynamic and thermoreversible nature of ionic crosslinks dominates the permanent covalent bonds.

## Figures and Tables

**Table 1:** The typical compounding formulation for the preparation of thermoplastic vulcanizates.

Ingredients	EPDM/HA60D (60/40) Loading (phr) ^a^	EPDM-*g*-MAH/HA60D (60/40) Loading (phr) ^a^	EPDM-*g*-MAH/HA60D (70/30) Loading (phr) ^a^
EPDM	100	-----	-----
EPDM-*g*-MAH	-----	100	100
HA60D	66.7	66.7	42.8
Zinc oxide	5.0	5.0	5.0
Stearic acid	1.0	1.0	1.0
MBTS ^b^	0.5	0.5	0.5
TMTD ^c^	1.0	1.0	1.0
Sulfur	1.5	1.5/2.0/3.0	1.5

^a^ Parts per hundred rubber. ^b^ 2-2′-Dithiobis(benzothiazole). ^c^ Tetramethylthiuram disulfide.

**Table 2:** The thermal properties of pure EPDM, EPDM-*g*-MAH, HA60D, and the EPDM/HA60D, EPDM-*g*-MAH/HA60D vulcanizates.

Sample (wt%)	*T_g_* (°C)	*T_m_* (°C)	
		First	Second
**EPDM**	−47	22	
**EPDM-*g*-MAH**	−55	162	
**HA60D**	26	82	
**EPDM/HA60D (60/40)**	−42	84	
**EPDM-*g*-MAH/HA60D (60/40)**	−51	81	166
**EPDM-*g*-MAH/HA60D (70/30)**	−51	82	164

**Table 3:** The self-healing properties of EPDM-*g*-MAH/HA60D and EPDM/HA60D vulcanizates.

Sample (wt%)	Self-Healing Property (%)
**EPDM-*g*-MAH/HA60D (60/40)**	87.4
**EPDM-*g*-MAH/HA60D (70/30)**	88.6
**EPDM/HA60D (60/40)**	----

## Data Availability

The data presented in this study are available upon request from the corresponding author.
